# Supplementary material for: Inhibition mechanism of SARS-CoV-2 main protease by ebselen and its derivatives
Source: Nat Commun. 2021 May 24;12:3061. doi: 10.1038/s41467-021-23313-7 (PMC8144557; doi:10.1038/s41467-021-23313-7)
Supplement: Supplementary file 1 — Supplementary Information [file 41467_2021_23313_MOESM1_ESM.pdf]

Supplementary Information

Inhibition mechanism of SARS-CoV-2 main protease by ebselen and its derivatives

Kangsa Amporndanai[1], Xiaoli Meng[2], Weijuan Shang[3], Zhenmig Jin[4], Michael Rogers,[5] Yao Zhao[4], Zihe Rao[4], Zhi-Jie Liu[6], Haitao Yang [4]*, Leike Zhang[3]*, Paul M. O'Neill[5]* and S. Samar Hasnain[1*].

[1] Molecular Biophysics Group, Department of Biochemistry and System Biology, Institute of System, Molecular and Integrative Biology, Faculty of Health and Life Sciences, University of Liverpool, Liverpool, L69 7ZB, United Kingdom.

[2] Department of Molecular and Clinical Pharmacology, Institute of Translational Medicine, Faculty of Health and Life Sciences, University of Liverpool, Liverpool, L69 3BX, United Kingdom.

[3] State Key Laboratory of Virology, Wuhan Institute of Virology, Chinese Academy of Sciences, Wuhan, Hubei, 430071, China

[4] Shanghai Institute for Advanced Immunochemical Studies and School of Life Science and Technology, ShanghaiTech University, Shanghai 201210, China.

[5] Department of Chemistry, Faculty of Science and Engineering, University of Liverpool, Liverpool, L69 7ZD, United Kingdom.

[6] iHuman Institute and School of Life Science and Technology, ShanghaiTech University, Shanghai 201210, China.

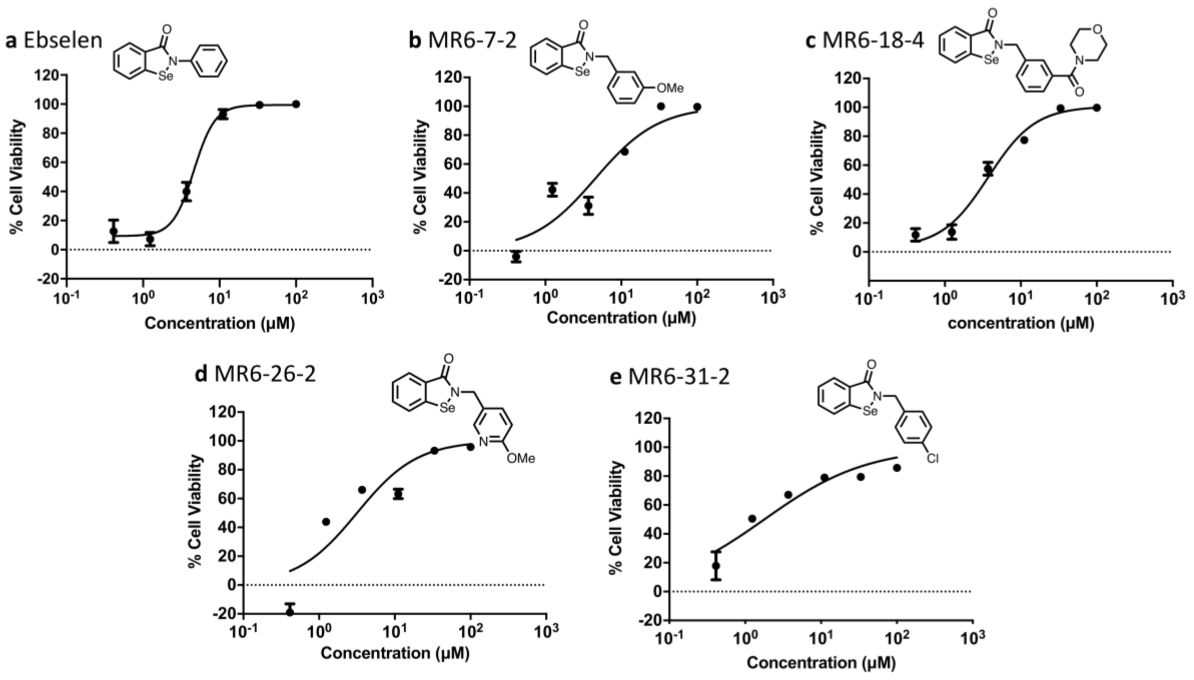

Supplementary Fig. 1 ***In vitro*** **EC_{50} curves of Vero E6 cells infected SARS-CoV-2 in lead compounds.** Cell viability percentage plots are means of *n*=3 measurements obtained over three independent experiments and error bars representing the standard error of the mean.

Supplementary Results and Discussion

Molecular docking of ebselen-based compounds to M^{pro}

Ebselen was docked to the catalytic site of wild-type and C145A M^{pro} (Supplementary Fig. 2). Docking solution with highest fitness score showed ebselen covalently binds to Cys145 of wild-type M^{pro} and establishes hydrophobic contacts between its phenyl tail and the side chains of Met49 and Met165. The selenium atom in docking solution is shifted 3Å away from the selenium observed in crystal structure of M^{pro}-ebselen and located closer to Asn142. In C145A mutant, we observed 180° flipped head group in docking solution due to absence of covalent bond formation. Significantly lower PLP fitness score in C145A mutant predicts *in silico* weaker affinity and confirms that Cys145 is the key residue for ebselen binding to M^{pro} catalytic pocket.

To describe structure-activity relationship of ebselen-based compounds, we compared PLP fitness scores of molecular docking to wild-type M^{pro} in our previous report[1] and IC_{50}s measured in this work (Supplementary Table 1). The methylene linker compounds (MR6-7-2, MR6-18-4 and MR6-26-2) have better fitness scores that correspond to stronger enzyme inhibition. Moreover, electron donating group like methoxy can enhance greater

affinity when incorporated with aromatic tail group (MR6-7-2 and MR6-26-2) while halogen substituent in MR6-31-2 shows the opposite aspect. The bigger moiety of carbonyl morpholine could increase inhibition level in the compound with methylene linker (MR6-18-4) but does not demonstrate the same effect in *N*-aryl compound (MR6-17-1). Thus, the enhancement of inhibition level of an ebselen-based compound could be achieved mainly through effectiveness of delivery of the compound to the catalytic pocket.

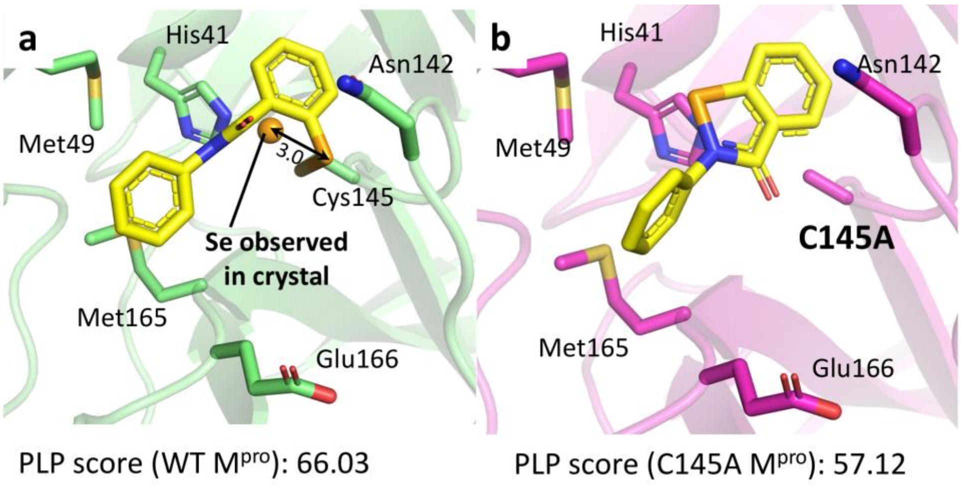

Supplementary Fig. 2 Ebselen docking to SARS-CoV-2 M^{pro} catalytic pocket. a Wild-type M^{pro}. **b** C145A mutant. Ebselen molecule is illustrated as yellow sticks. Amino acid residues of wild-type and C145A M^{pro} are shown as light green and magenta sticks, respectively. Selenium atom observed in crystal structure of M^{pro}-ebselen is shown as orange sphere.

Supplementary Table 1. The average PLP fitness for the compounds accessed for wild-type M^{pro}. Fitness scores are taken from our previous work[1].

Compound	Average PLP fitness score (wild-type M^{pro})	IC$_{50}$ (wild-type M^{pro})
Ebselen	66.03	670 nM
MR6-7-2	79.75	363 nM
MR6-17-1	79.42	702 nM
MR6-18-4	75.84	345 nM
MR6-26-2	79.89	467 nM
MR6-31-2	69.15	824 nM

LC-MS/MS analysis to determine covalent binding of ebselen to proteins

To characterise the covalent adducts formed by ebselen on proteins, human glutathione S-transferase pi (GSTP) was chosen as a model as it contains several reactive cysteine residues. His-GSTP captured on nickel beads was incubated with 1 mM ebselen in ammonium bicarbonate buffer (50 mM, pH 7). LC-MS/MS analysis of the tryptic digest of ebselen treated GSTP revealed a cysteine-ebselen adduct with a mass addition of 274.996 amu. Supplementary Fig. 3a shows a representative MS/MS spectrum for a doubly charged ion at m/z 677.772, corresponding to the tryptic peptide ^{45}ASCLYGQLPK^{54} with an additional mass of 274.996 amu. The peptide sequence was confirmed by partial singly charged y and b series ions. The presence of an abundant ion of m/z 275.987, which corresponds to the molecular ion of ebselen after cleavage of sulphur-selenium bond and a fragment ion derived from ebselen (m/z 196.071) provided further evidence of the modification.

The binding of ebselen to M^{pro} appeared to be variable. Ebselen was previously reported to covalently bind to Cys145 in M^{pro} peptide FTIKGSFLNGSCGSVGF with a mass addition of 275 amu[3]. However, this adduct was not detected on Cys145 in this study, which is consistent with the finding in the structural study. The proposed hydrolysis reaction could result in an adduct with a mass addition of 79.99 amu where selenium is attached to Cys145. The peptide containing this adduct could potentially form a cross-linked peptide with another cysteine containing peptide, which is generally difficult to be identified by searching software. Interestingly, ebselen was found to covalently bind to Cys16 with a mass addition of 274.996 amu, indicating this adduct is stable without the presence of neighbouring catalytic histidine residues nearby (Supplementary Fig. 3b).

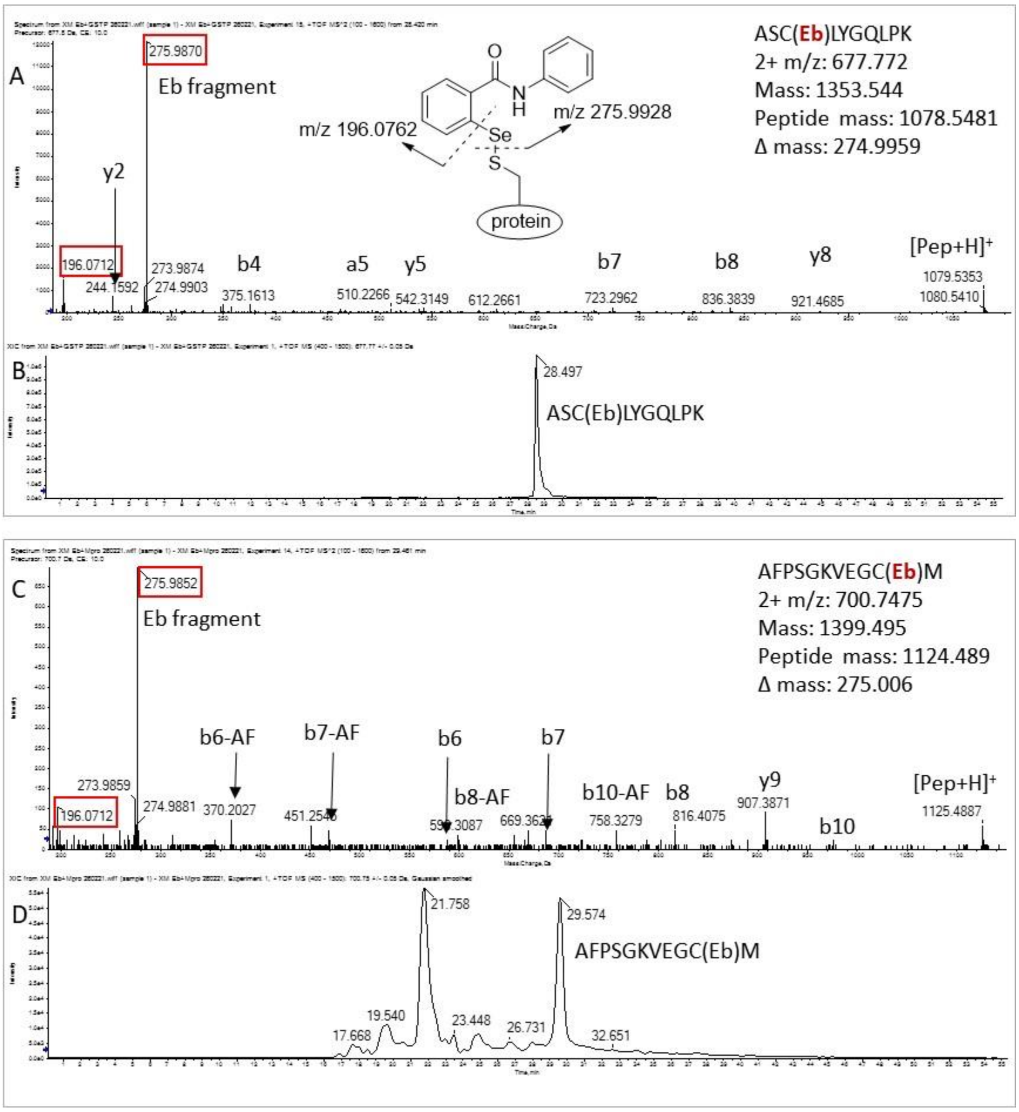

Supplementary Fig. 3 LC-MS/MS analysis of ebselen protein modification identified *in vitro*. a MS/MS spectrum showing peptide ^{45}ASCLYGQLPK^{54} in human glutathione S-transferase pi (accession: P09211) was modified by ebselen at Cys47 with a mass addition of 274.996 amu. **b** Extracted ion corresponding to ebselen modified peptide ^{45}ASCLYGQLPK^{54}. **c** MS/MS spectrum show peptide ^{7}AFPSGKVEGCM^{17} in M^{pro} (accession: P0DTD1) was modified by ebselen at Cys16 with a mass addition of 274.996 amu. **d** Extracted ion corresponding to ebselen modified peptide ^{7}AFPSGKVEGCM^{17}.

Supplementary Methods

Molecular docking

The small molecule 3D structures were built using Spartan '14 v1.1.8 and energy minimised using MMFF forcefield. Molecular docking of the small molecules into the active site of M^{Pro} was performed using GOLD Suite v5.21. The crystal structure of ligand-free M^{Pro} (PDB: 7BAJ) was prepared for use in docking calculations by addition of hydrogen atoms in the protein located in the active site. The active site for docking was defined to be selecting sulphur atom of the Cysteine 145 residue for covalent docking to wild-type M^{pro}. Each ligand was set to undergo 10 GA runs with no early termination allowed, lone pairs were not saved, and all solutions were kept. The rest of the settings were left as default. ChemPLP fitness scores were the output obtained. All docked ligands and amino acid residues were visualised using Pymol software.

Modification of proteins by ebselen

His-GSTP was expressed in *E.coli* as described previously[2]. Purified His-GSTP captured on nickel beads was incubated with 1 mM ebselen in ammonium bicarbonate buffer (50 mM, pH 7) for 16 h. The beads were then washed 5 times with 1000 µL ammonium bicarbonate buffer. The protein was subjected to on-bead tryptic digestion. In brief, a suspension of beads in 30 µL of 50 mM ammonium bicarbonate buffer was incubated with 20 ng of trypsin for 16 h at 37 °C and the digest was analysed by LC-MS/MS. In addition, 1 mM ebselen was incubated with purified M^{pro} in ammonium bicarbonate buffer (50 mM, pH 7) for 16 h. The protein was precipitated by adding 4 volumes of ice-cold acetone. After centrifugation at 14,000 g for 20 min., the pellet was resuspended in in ammonium bicarbonate buffer and digested with chymotrypsin (protein enzyme ratio: 100 to 1) at 37°C for 16h. The digest was analysed by LC-MS/MS.

LC-MS/MS analysis of ebselen protein adducts

The digests were analysed by a Triple TOF 6600 mass spectrometer (Sciex). Samples were reconstituted in 50 µL 0.1% formic acid and 2 µL of samples were delivered into the instrument using an Eksigent Nano-LC system mounted with a nanoACQUITY UPLC Symmetry C18 Trap Column and an analytical BEH C18 nanoACQUITY Column (Waters, MA, USA). A NanoSpray III source was fitted with a 10 µm inner diameter PicoTip emitter (New Objective). Samples were loaded in 0.1% formic acid onto the trap, which was then washed with 2% ACN/0.1% FA for 10 min at 2 µL/min before switching in-line with the analytical column. A gradient of 2−50% (v/v) ACN/0.1% (v/v) FA over 90 min was applied to the column at a flow rate of 300 nL/min. Spectra were

acquired automatically in positive ion mode using information-dependent acquisition, using mass ranges of 400−1600 amu in MS and 100−1400 amu in MS/MS. Up to 25 MS/MS spectra were acquired per cycle (approximately 10 Hz) using a threshold of 100 counts per s, with dynamic exclusion for 12 s and rolling collision energy.

Analysis of ebselen modified proteins

LC-MS/MS data were searched against the reviewed human proteome (UniProt/SwissProt accessed October 2020), using ProteinPilot software, v5.0 (Sciex). Data were refined using default parameters and searches performed with the following parameters: for ebselen modified GSTP, enzymatic cleavage restriction for trypsin, variable modifications (methionine oxidation, +15.99, asparagine and glutamine deamidation, +0.98), and ebselen modification of cysteine (+274.996); for ebselen modified M^{pro}, enzymatic cleavage restriction for chymotrypsin, variable modifications (methionine oxidation, +15.99, asparagine and glutamine deamidation, +0.98), and ebselen modification of cysteine (+274.996 or 79.99).

Synthesis of ebselen derivatives

General Procedure 1: Amide preparation

To a stirring solution of the required carboxylic acid (1 eq) in anhydrous DCM (0.2M) was added oxalyl chloride (2 eq) and catalytic DMF (3-4 drops). The resulting reaction mixture was stirred at room temperature for 1.5 hours. The reaction mixture was then concentrated under reduced pressure and the resulting acid chloride intermediate dried thoroughly under high-vacuum. The acid chloride was re-dissolved in anhydrous DCM (0.2M), followed by the selected amine (1-4 eq) and NEt_3 (2-3 eq). The reaction mixture was left to stir overnight or until acid chloride was completely consumed under an N_2 environment. The reaction mixture was diluted with DCM and washed with distilled water, 1M HCl, sat. NaHCO3 and brine. The organic phase was dried over anhydrous $MgSO_4$, filtered and concentrated under reduced pressure. The crude products were purified by FCC (flash chromatography) if required.

General procedure 2: Benzisoselenazolone formation via CuI ring closure

A solution of copper iodide (0.2-1.0 eq) and 1,10-phenanthroline (0.2-1.0 eq) in DMF (30 mL/g) was stirred under nitrogen for 15 minutes (dark orange/brown solution). The required amide (1 eq), selenium (1.2 eq) and potassium carbonate (1.5 eq) were then added before heating to 110°C for 36 hours under a nitrogen atmosphere. The reaction mixture was then stirred in brine for 3 hours before extracting with ethyl acetate (3 x 30mL). The combined

organic extracts were then dried over anhydrous $MgSO_4$, filtered and concentrated under reduced pressure. The crude products were purified by FCC.

General procedure 3: amide coupling via HATU coupling conditions

To a solution of the desired acid (1 eq) in anhydrous DMF (0.2M) was added the desired amine (1.1 eq), HATU (1.6 eq) and DIPEA (3-4 eq). The resulting reaction mixture was stirred at room temperature overnight. EtOAc was added to the reaction mixture and washed with sat. $NaHCO_3$ (x3), water and brine. The organic phase was then dried over $MgSO_4$, filtered and concentrated under reduced pressure. The crude products were purified by FCC.

General procedure 4: Benzisoselenazolone formation with KO^{t}Bu

To a solution of selenium (1.3 eq) in anhydrous DMF (0.2M) was added potassium *tert*-butoxide (1.6 eq) at 0°C. The reaction mixture was allowed to warm to room temperature for 15 minutes before the desired amide (1 eq) was added and the resulting reaction mixture was heated to 130°C overnight. The reaction mixture was cooled to room temperature and added to a stirred solution of brine for 3 hours and extracted with EtOAc (x3). The combined organic phases were dried over $MgSO_4$, filtered and concentrated under reduced pressure. The crude products were purified by FCC.

General procedure 5: Boc deprotection

To a solution of a given Boc protected amine (1.0 eq) in TFA:DCM (0.2M, 1:5) was allowed to stir at room temperature overnight. The reaction mixture was neutralised with sat. $NaHCO_3$, and extracted with DCM (x 3). The organic phase was dried over $MgSO_4$, filtered and concentrated under reduced pressure to give the free amine species.

General procedure 6: Benzisoselenazolone formation via reaction with dichloride

To a solution of 2, 2'-diselanediyldibenzoyl chloride (1 eq) in anhydrous DCM (0.2M) was added a given amine (1.1-2 eq) and NEt3 (2-3 eq). The reaction mixture was stirred at room temperature until complete consumption of starting material before concentrated under reduced pressure and the crude mixture was taken up in H_2O and stirred for 10-15 minutes then filtered. If required, the crude product was extracted with EtOAc (3 x) then dried over anhydrous $MgSO_4$, filtered and concentrated under reduced pressure. The crude products were purified by FCC (flash chromatography) if required.

Synthesis of MR6-7-2

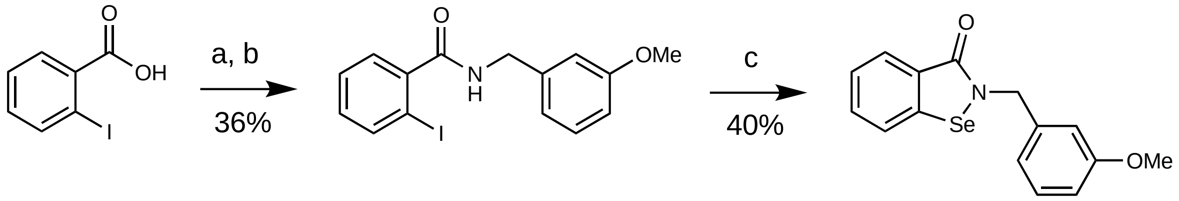

Supplementary Scheme 1. Synthetic route of MR6-7-2. (a) oxalyl chloride, DCM, DMF (10 mol%), rt, 2 hrs; (b) 3-methoxy-benzylamine, NEt_3, DCM, rt, 16 hrs; (c) CuI, 1,10-phenanthroline, Se powder, K_2CO_3, DMF, 110°C.

2-Iodo-*N*-(3-methoxybenzyl)benzamide

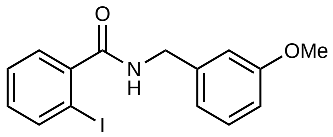

General procedure 1 was followed using 2-iodobenoic acid (1.08 g, 4.034 mmol) and 3-methoxy benzylamine (0.57 ml) to give the title compound as a white solid (0.52 g, 36% yield): ^{1}H NMR (500 MHz, $CDCl_3$) δ = 7.82 (ap d, *J* = 8.1 Hz, 1H), 7.38-7.30 (m, 2H), 7.24 (ap t, *J* = 8.1 Hz, 1H), 7.08-7.04 (m, 1H), 6.95-6.92 (m, 2H), 6.81 (dd, *J* = 8.1, 2.5 Hz, 1H), 6.45 (br s, NH), 4.55 (d, *J* = 5.8 Hz, 2H), 3.79 (s, 3H). ^{13}C NMR (126 MHz, $CDCl_3$) δ = 169.3, 159.9, 139.9, 139.3, 131.1, 129.7, 128.3, 128.1, 120.3, 113.5, 113.3, 92.6, 55.3, 44.1. HRMS (ES+, *m/z*) calculated for $C_{15}H_{14}INO_2Na$ $[M+Na]^+$ 359.9961, found 389.9976. Diff = -3.85 ppm.

2-(3-Methoxybenzyl)benzo[d][1,2]selenazol-3(2H)-one (MR6-7-2)

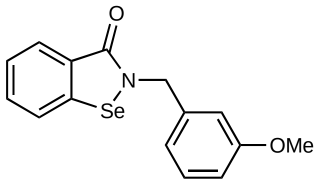

General procedure 2 was followed using 2-iodo-*N*-(3-methoxybenzyl)benzamide (0.50 g, 1.48 mmol) to give the title compound as a yellow solid (0.02 g, 40% yield): ^{1}H NMR (400 MHz, $CDCl_3$) δ = 8.07 (ap d, *J* = 7.9 Hz, 1H), 7.60-7.54 (unresolved d, 2H), 7.45-7.39 (m, 1H), 7.32-7.23 (m, 1H), 6.98-6.92 (m, 1H), 6.90-6.84 (m, 2H), 4.98 (s, 2H), 3.79 (s, 3H). ^{13}C NMR (100 MHz, $CDCl_3$) δ = 167.2, 160.0, 138.7, 138.1, 132.0, 129.9, 128.9, 127.4, 126.2, 124.0, 120.8, 114.0, 113.9, 55.3, 48.6. HRMS (ES+, *m/z*) calculated for $C_{15}H_{13}NO_2{}^{80}SeNa$ $[M+Na]^+$ 342.0004 found 342.0007. Diff = -0.97 ppm.

Synthesis of MR6-26-2

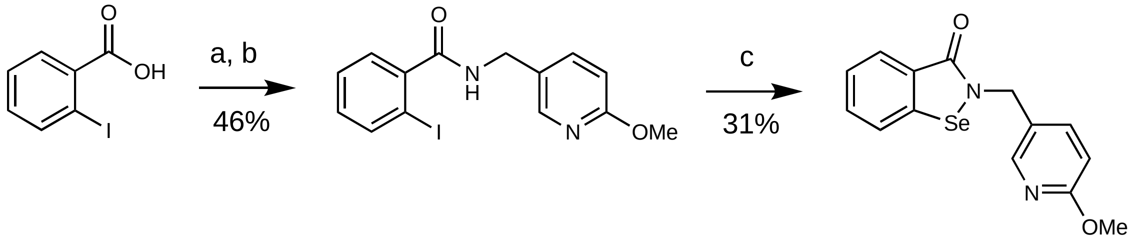

Supplementary Scheme 2. Synthetic route of MR6-26-2. (a) oxalyl chloride, DCM, DMF (10 mol%), rt, 16 hrs; (b) (6-methoxy-pyridin-3yl)-methanamine, NEt_3, DCM, rt, 16 hrs; (c) Se, KO*t*Bu, DMF, 130°C, 16 hrs.

2-Iodo-*N*-((6-methoxypyridin-3-yl)methyl)benzamide

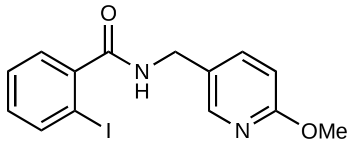

General procedure 1 was followed using 2-iodobenoic acid (0.32 g, 1.29 mmol) and (6-methoxypyridin-3-yl)methanamine dihydrochloride (0.3 g) to give the title compound as a brown solid (0.35 g, 46% yield): ^{1}H NMR (500 MHz, $CDCl_3$) δ = 8.06 (d, *J* = 2.3 Hz, 1H), 7.76 (d, *J* = 8.0 Hz, 1H), 7.60 (dd, *J* = 8.4, 2.3 Hz, 1H), 7.29-7.23 (m, 2H), 7.05-6.98 (m, 1H), 6.75 (br s, NH), 6.66 (d, *J* = 8.4 Hz, 1H), 4.43 (d, *J* = 5.9 Hz, 2H) 3.87 (s, 3H). ^{13}C NMR (100 MHz, $CDCl_3$) δ = 169.4, 163.7, 146.3, 141.8, 139.8, 139.1, 131.1, 128.2, 128.1, 126.3, 110.8, 92.5, 53.5, 40.9. HRMS (ES+, *m/z*) calculated for $C_{14}H_{14}IN_2O_2$ $[M+H]^+$ 369.0094, found 369.0098. Diff = -1.02 ppm.

2-((6-Methoxypyridin-3-yl)methyl)benzo[*d*][1,2]selenazol-3(2*H*)-one (MR6-26-2)

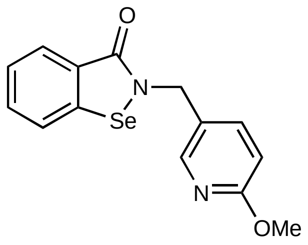

General procedure 4 was followed using 2-Iodo-*N*-((6-methoxypyridin-3-yl)methyl)benzamide (0.15 g, 0.41 mmol) to give the title compound as a white solid (0.04 g, 31% yield): ^{1}H NMR (500 MHz, $CDCl_3$) δ = 8.18 (d, *J* = 2.0 Hz, 1H), 8.07 (d, *J* = 8.0 Hz, 1H), 7.62-7.57 (m, 3H), 7.47-7.44 (m, 1H), 6.74 (d, *J* = 8.5 Hz, 1H), 4.95 (br s, 2H) 3.95 (s, 3H). ^{13}C NMR (100 MHz, $CDCl_3$) δ = 167.2, 164.3, 146.7, 139.3, 137.9, 132.1, 128.9, 127.3, 126.3, 125.8, 124.1, 111.4, 53.4, 45.5. HRMS (ES+, *m/z*) calculated for $C_{14}H_{13}N_2O_2{}^{80}Se$ $[M+H]^+$ 321.0137 found 321.014. Diff = -0.95 ppm.

Synthesis of MR6-31-2

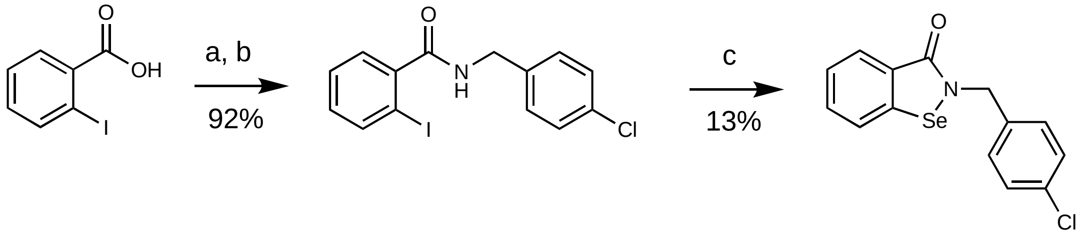

Supplementary Scheme 3. Synthetic route of MR6-31-2. (a) oxalyl chloride, DCM, DMF (10 mol%), rt, 2 hrs; (b) 4-chloro-benzylamine, NEt_3, DCM, rt, 16 hrs; (c) CuI, 1,10-phenanthroline, Se powder, K_2CO_3, DMF, 110ºC.

***N*-(4-Chlorobenzyl)-2-iodobenzamide**[4]

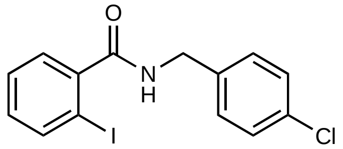

General procedure 1 was followed using 2-iodobenzoic acid and 4-chlorobenzylamine to give the title compound as a white solid (644 mg, 92% yield): 1H NMR (400 MHz, $CDCl_3$) δ = 7.86 (d, *J* = 8 Hz, 1H), 7.42 – 7.30 (m, 6H), 7.10 (td, *J* = 8, 2 Hz, 1H), 6.07 (s, 1H), 4.61 (d, *J* = 6 Hz, 1H). ^{13}C NMR (101 MHz, $CDCl_3$) δ = 169.4, 142.0, 140.1, 136.3, 133.7, 131.5, 129.7, 129.1, 128.5, 128.4, 92.5, 43.6. HRMS (CI, *m/z*) calculated for $C_{14}H_{12}ClINO$ [M+H]+ 371.9652, found 371.9657.

2-(4-Chlorobenzyl)benzo[*d*][1,2]selenazol-3(2*H*)-one (MR6-31-2)

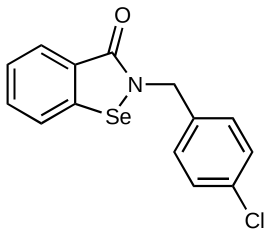

General procedure 2 was followed using *N*-(4-chlorobenzyl)-2-iodobenzamide to give the title compound as a white solid (75 mg, 13% yield). ^{1}H NMR (400 MHz, $CDCl_3$) δ = 8.07 (d, *J* = 8 Hz, 1H), 7.59 (d, *J* = 4 Hz, 2H), 7.46 – 7.41 (m, 1H), 7.35 – 7.27 (m, 4H), 4.98 (s, 2H). ^{13}C NMR (101 MHz, $CDCl_3$) δ = 167.4, 166.6, 138.0, 135.8, 134.4, 132.3, 129.9, 129.4, 129.2, 129.1, 126.5, 124.2, 48.0. HRMS (CI, *m/z*) calculated for $C_{14}H_{11}ClNOSe$ [M+H]+ 323.9694, found 323.9689. Elemental analysis calculated for $C_{14}H_{10}ClNOSe$: C, 52.12; H, 3.12; N, 4.34. Found: C, 52.22; H, 3.07; N, 4.21. IR (neat) ν_{max}/cm^{-1} 3052 (m, C-H aromatic), 2908 (m, C-H aliphatic), 1634 (s, C=O amide), 1586 (s, C-C aromatic), 1443 (m, C-H aliphatic) cm^{-1}. Melting Point: 118-120°C.

Synthesis of MR6-17-1

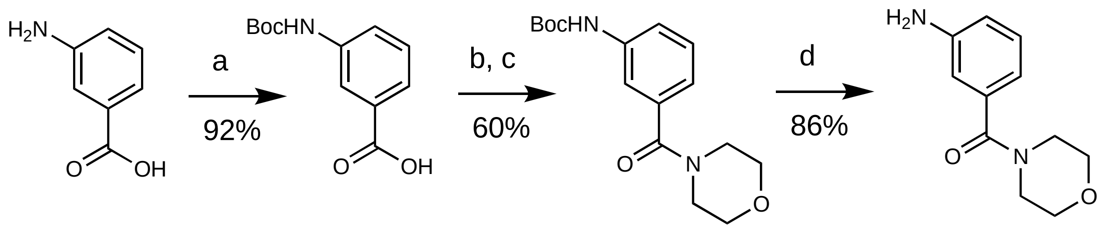

Supplementary Scheme 4. Preparation of (3-aminophenyl)(morpholino)methanone. (a) Boc anhydride, DCM, NMe_3, rt, 16 hrs; (b) oxalyl chloride, DCM, DMF (10 mol%), rt, 2 hrs; (c) Morpholine, NEt_3, DCM, rt, 16 hrs; (d) TFA, DCM, rt, 16 hrs.

Boc-3-aminobenzoic acid

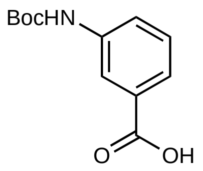

To a solution of 3-aminobenzoic acid (2 g, 16.33 mmol) in DCM (82 ml) was added di-*tert*-butyl dicarbonate (7.13 g, 32.66 mmol, 2.0 eq) and dropwise addition of trimethylamine (2.5 ml, 18 mmol, 1.1 eq). The reaction mixture was allowed to stir overnight before dilution with DCM (100 ml) and washed with distilled water (3 x 150 ml). The combined organic phases were dried over $MgSO_4$ and concentrated under reduced pressure to give the titled compound as a white powder (3.58 g, 92% yield): ^{1}H NMR (500 MHz, DMSO) δ = 12.90 (br s, OH), 9.55 (s, 1H), 8.16 (s, 1H), 7.63 (d, *J* = 8.0 Hz, 1H), 7.55(d, *J* = 8.0 Hz, 1H), 7.37 (ap t, *J* = 7.8 Hz, 1H), 1.48 (s, 9H). ^{13}C NMR (100 MHz, DMSO) δ = 167.8, 153.2, 140.3, 131.7, 129.3, 123.4, 122.7, 119.2, 79.8, 28.6.

HRMS (ES-, *m/z*) calculated for $C_{12}H_{14}NO_4$ $[M-H]^-$ 236.0928 found 236.093. Diff = -0.85 ppm.

Boc-3-(morpholine-4-carbonyl)phenyl)carbamate

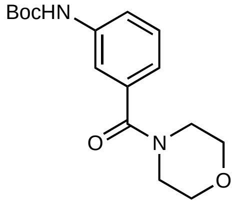

General procedure 1 was followed using Boc-3-aminobenzoic acid (2.0 g, 8.43 mmol) and morpholine (1.10 ml) to give the title compound as an off-white solid (1.56 g, 60% yield): ^{1}H NMR (500 MHz, $CDCl_3$) δ = 7.49 (br s, 1H), 7.43 (ap d, *J* = 8.0 Hz, 1H), 7.31 (ap t, *J* = 8.0 Hz, 1H), 7.04 (ap d, *J* = 8.0 Hz, 1H), 6.93 (br s, NH), 3.83-

3.58 (unresolved m, 6H), 3.53-3.42 (unresolved m, 2H), 1.52 (s, 9H). ^{13}C NMR (126 MHz, $CDCl_3$) δ = 170.1, 152.7, 138.9, 136.0, 129.2, 121.3, 119.9, 117.3, 80.9, 66.9 (4C), 28.3. HRMS (ES+, *m/z*) calculated for $C_{16}H_{22}N_2O_4Na$ $[M+Na]^+$ 329.1472, found 329.1476. Diff = -1.31 ppm.

(3-Aminophenyl)(morpholino)methanone

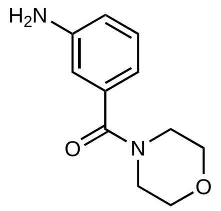

General procedure 5 was followed using Boc-3-(morpholine-4-carbonyl)phenyl)carbamate (0.95 g, 3.10 mmol) to give the title compound as a colourless oil (0.55 g, 86% yield): ^{1}H NMR (500 MHz, $CDCl_3$) δ = 7.17 (app td, *J* = 7.7, 0.9 Hz, 1H), 6.74-6.73 (m, 1H), 6.73-6.72 (m, 1H), 6.71 (s, 1H), 3.83-3.58 (unresolved m, 6H), 3.53-3.42 (unresolved m, 2H). ^{13}C NMR (126 MHz, $CDCl_3$) δ = 170.6, 146.8, 136.4, 129.4, 116.7, 116.3, 113.4, 66.9 (4C). HRMS (ES+, *m/z*) calculated for $C_{11}H_{15}N_2O_2$ $[M+H]^+$ 207.1128, found 207.1128. Diff = -0.2 ppm.

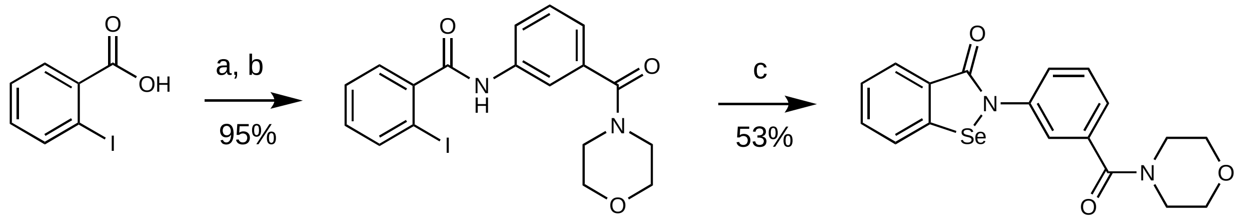

Supplementary Scheme 5. Synthetic route of MR6-17-1. (a) oxalyl chloride, DCM, DMF (10 mol%), rt, 2 hrs; (b) (3-aminophenyl)(morpholino)methanone, NEt_3, DCM, rt, 16 hrs; (c) Se, KO^{t}Bu, DMF, 130ºC, 16 hrs.

2-Iodo-*N*-(2-(morpholine-4-carbonyl)phenyl)benzamide

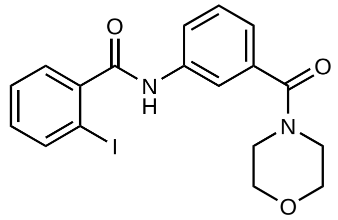

General procedure 1 was followed using 2-iodobenoic acid (2.64 g, 10.6 mmol) and (3-Aminophenyl)(morpholino)methanone (2.41 g) to give the title compound as a light brown solid (4.40 g, 95% yield): ^{1}H NMR (500 MHz, $CDCl_3$) δ = 8.14 (br s, 1H), 7.91 (d, *J* = 8.2 Hz, 1H), 7.77 (br d, *J* = 8.2 Hz, 1H), 7.72

(br s, 1H), 7.50 (dd, *J* = 7.5, 1.3 Hz, 1H), 7.46-7.38 (m, 2H), 7.19-7.14 (m, 2H), 3.75-3.45 (unresolved m, 8H). ^{13}C NMR (100 MHz, $CDCl_3$) δ = 169.8, 167.5, 141.9, 140.0, 138.1, 135.9, 131.6, 129.3, 128.6, 128.3, 123.2, 121.7, 119.3, 92.4, 66.9 (4C). HRMS (ES+, *m/z*) calculated for $C_{18}H_{17}IN_2O_3Na$ $[M+Na]^+$ 459.0176, found 459.0176. Diff = 0.04 ppm.

2-(3-(Morpholine-4-carbonyl)phenyl)benzo[*d*][1,2]selenazol-3(2H)-one (MR6-17-1)

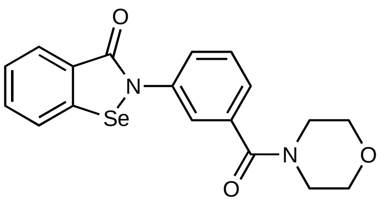

General procedure 4 was followed using 2-iodo-*N*-(2-(morpholine-4-carbonyl)phenyl)benzamide (0.37 g, 0.85 mmol) to give the title compound as a white solid (0.17 g, 53% yield): ^{1}H NMR (400 MHz, $CDCl_3$) δ = 8.02 (d, *J* = 7.7 Hz, 1H), 7.67-7.65 (m, 1H), 7.63 (s, 1H), 7.61-7.56 (m, 2H), 7.43-7.38 (m, 2H), 7.25 (br d, *J* = 7.7 Hz, 1H), 3.75-3.45 (unresolved m, 8H). ^{13}C NMR (100 MHz, $CDCl_3$) δ = 169.3, 165.9, 139.4, 137.7, 136.3, 132.8, 129.7, 129.3, 127.3, 126.7, 126.6, 125.2, 124.0, 123.9, 66.9 (4C). HRMS (ES+, *m/z*) calculated for $C_{18}H_{16}N_2O_3{}^{80}SeNa$ $[M+Na]^+$ 411.0218, found 411.0223. Diff = -1.13 ppm.

Synthesis of MR6-18-4

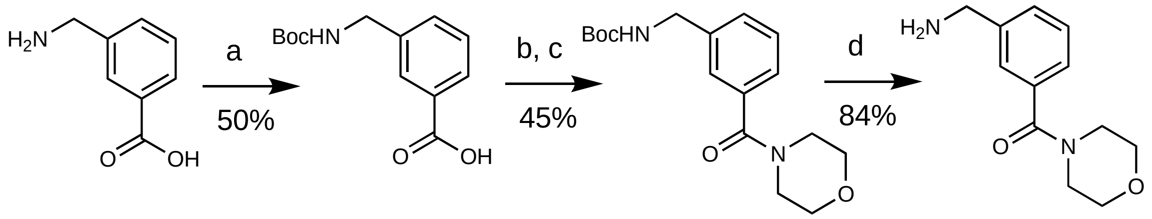

Supplementary Scheme 6. Preparation of (3-(aminomethyl)phenyl)(morpholino)methanone. (a) Boc anhydride, DCM, NMe_3, rt, 16 hrs; (b) oxalyl chloride, DCM, DMF (10 mol%), rt, 2 hrs; (c) Morpholine, NEt_3, DCM, rt, 16 hrs; (d) TFA, DCM, rt, 16 hrs.

***tert*-Butyl (3-(morpholine-4-carbonyl)benzyl)carbamate**

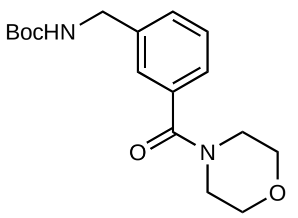

General procedure 1 was followed using 3-(((*tert*-butoxycarbonyl)amino)methyl)benzoic acid (0.91 g, 1.7 mmol) and morpholine (0.41 ml) to give the title compound as a white solid (0.58 g, 50% yield): ^{1}H NMR (500 MHz, $CDCl_3$) δ = 7.20-7.14 (m, 3H), 7.11 (br d, *J* = 7.2 Hz, 1H), 5.60 (br s, NH), 4.12 (d, *J* = 5.0 Hz, 2H), 3.65-3.40 (unresolved m, 6H), 3.33-3.19 (unresolved m, 2H), 1.29 (s, 9H). ^{13}C NMR (126 MHz, $CDCl_3$) δ = 170.1, 156.0, 139.9, 135.3, 128.6, 128.5, 125.8, 125.6, 79.1, 66.6 (4C), 44.0, 28.3. HRMS (ES+, *m/z*) calculated for $C_{17}H_{24}N_2O_4Na$ $[M+Na]^+$ 343.1628, found 343.1632. Diff = -0.94 ppm.

(3-(Aminomethyl)phenyl)(morpholino)methanone

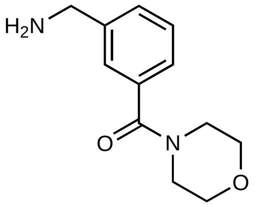

General procedure 5 was followed using *tert*-butyl (3-(morpholine-4-carbonyl)benzyl)carbamate (0.58 g, 1.7 mmol) to give the title compound as a white solid (0.18 g, 45% yield). The compound was carried through without characterisation into the next step

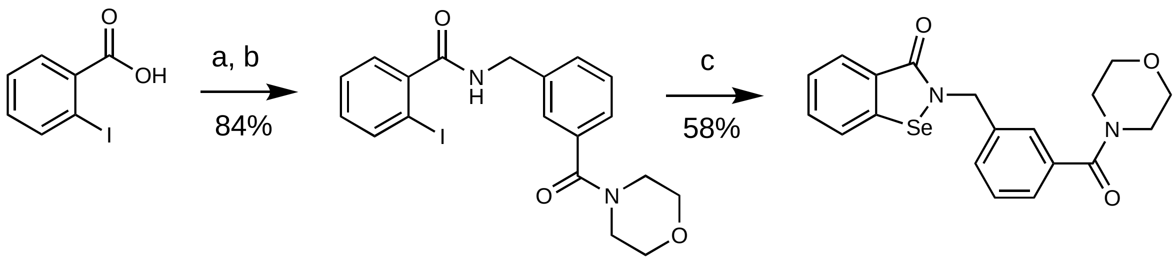

Supplementary Scheme 7. Synthetic route of MR6-18-4. (a) oxalyl chloride, DCM, DMF (10 mol%), rt, 2 hrs; (b) (3-(aminomethyl)phenyl)(morpholino)methanone, NEt_3, DCM, rt, 16 hrs; (c) Se, KO^{t}Bu, DMF, 130°C, 16 hrs.

2-Iodo-*N*-(3-(morpholine-4-carbonyl)benzyl)benzamide

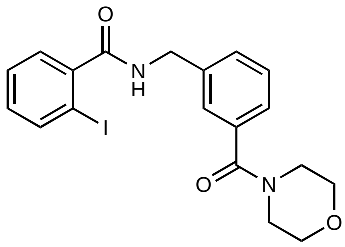

General procedure 1 was followed using 2-iodobenoic acid (0.18 g, 6.33 mmol) and (3-(Aminomethyl)phenyl)(morpholino)methanone (0.24 g) to give the title compound as a light brown solid (0.28 g, 84% yield): ^{1}H NMR (500 MHz, $CDCl_3$) δ = 7.85 (d, *J* = 7.6 Hz, 1H), 7.45 (d, *J* = 7.6 Hz, 1H), 7.41 (br s, 1H), 7.39-7.34 (m, 3H), 7.26 (br d, *J* = 7.6 Hz, 1H), 7.10 (dt, *J* = 7.6, 2.5 Hz, 1H), 6.64 (br s, NH), 4.61 (d, *J* = 6.2 Hz, 2H), 3.78-3.40 (unresolved m, 8H). ^{13}C NMR (100 MHz, $CDCl_3$) δ = 170.1, 169.4, 141.9, 139.9, 138.6, 135.7, 131.2, 129.5, 128.9, 128.3, 128.2, 126.6, 126.1, 92.5, 66.9 (4C), 43.7. HRMS (ES+, *m/z*) calculated for $C_{19}H_{19}IN_2O_3$ $[M+H]^+$ 451.0513, found 451.0513. Diff = -0.07 ppm.

2-(3-(Morpholine-4-carbonyl)benzyl)benzo[*d*][1,2]selenazol-3(2*H*)-one (MR6-18-4)

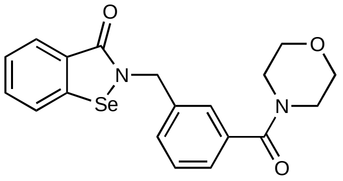

General procedure 4 was followed using 2-iodo-*N*-(3-(morpholine-4-carbonyl)benzyl)benzamide (0.17 g, 0.85 mmol) to give the title compound as an off-white solid (0.094 g, 58% yield): ^{1}H NMR (400 MHz, $CDCl_3$) δ = 8.07 (d, *J* = 7.7 Hz, 1H), 7.64-7.57 (m, 2H), 7.47-7.35 (m, 5H), 5.04 (br s, 2H), 3.80-3.38 (unresolved m, 8H). ^{13}C NMR (100 MHz, $CDCl_3$) δ = 169.9, 167.3, 134.0, 137.9, 135.9, 132.3, 129.7, 129.2, 129.0, 127.0, 126.9, 126.8, 126.4, 124.2, 66.8 (4C), 48.1. HRMS (ES+, *m/z*) calculated for $C_{19}H_{18}N_2O_3{}^{80}Se$ $[M+H]^+$ 403.0555, found 403.0553. Diff = 0.64 ppm.

Supplementary References

1. Amporndanai, K. *et al.* Novel Selenium-based compounds with therapeutic potential for SOD1-linked amyotrophic lateral sclerosis. *EBioMedicine* **59**, 102980 (2020).

2. Meng, X. *et al.* Abacavir Forms Novel Cross-Linking Abacavir Protein Adducts in Patients. *Chem. Res. Toxicol.* **27**, 524–535 (2014).

3. Jin, Z. *et al.* Structure of Mpro from SARS-CoV-2 and discovery of its inhibitors. *Nature* **582**, 289–293 (2020).
