# Supplementary material for: Inhibition mechanism of SARS-CoV-2 main protease by ebselen and its derivatives
Source: Nat Commun. 2021 May 24;12:3061. doi: 10.1038/s41467-021-23313-7 (PMC8144557; doi:10.1038/s41467-021-23313-7)
Supplement: Supplementary file 3 — Reporting Summary [file 41467_2021_23313_MOESM3_ESM.pdf]

nature research

Corresponding author(s): S. Samar Hasnain

Last updated by author(s): Mar 25, 2021

Reporting Summary

Nature Research wishes to improve the reproducibility of the work that we publish. This form provides structure for consistency and transparency in reporting. For further information on Nature Research policies, see our Editorial Policies and the Editorial Policy Checklist.

Statistics

For all statistical analyses, confirm that the following items are present in the figure legend, table legend, main text, or Methods section.

n/a	Confirmed	
☐	☒	The exact sample size (*n*) for each experimental group/condition, given as a discrete number and unit of measurement
☐	☒	A statement on whether measurements were taken from distinct samples or whether the same sample was measured repeatedly
☒	☐	The statistical test(s) used AND whether they are one- or two-sided<br>*Only common tests should be described solely by name; describe more complex techniques in the Methods section.*
☒	☐	A description of all covariates tested
☒	☐	A description of any assumptions or corrections, such as tests of normality and adjustment for multiple comparisons
☐	☒	A full description of the statistical parameters including central tendency (e.g. means) or other basic estimates (e.g. regression coefficient) AND variation (e.g. standard deviation) or associated estimates of uncertainty (e.g. confidence intervals)
☒	☐	For null hypothesis testing, the test statistic (e.g. *F*, *t*, *r*) with confidence intervals, effect sizes, degrees of freedom and *P* value noted<br>*Give P values as exact values whenever suitable.*
☒	☐	For Bayesian analysis, information on the choice of priors and Markov chain Monte Carlo settings
☒	☐	For hierarchical and complex designs, identification of the appropriate level for tests and full reporting of outcomes
☒	☐	Estimates of effect sizes (e.g. Cohen's *d*, Pearson's *r*), indicating how they were calculated

Our web collection on statistics for biologists contains articles on many of the points above.

Software and code

Policy information about availability of computer code

Data collection	Analyst (v1.5); Multi-Quant (v3.0, AB Sciex); GOLD Suite (v5.21)
Data analysis	Xia2 (v0.3.8.0); CCP4 (v7.1.010); Coot (v0.8.9.2); GraphPad Prism (v8.3.1); Microsoft Excel (v16.45); PyMOL (v2.4.0);

For manuscripts utilizing custom algorithms or software that are central to the research but not yet described in published literature, software must be made available to editors and reviewers. We strongly encourage code deposition in a community repository (e.g. GitHub). See the Nature Research guidelines for submitting code & software for further information.

Data

Policy information about availability of data

All manuscripts must include a data availability statement. This statement should provide the following information, where applicable:

- Accession codes, unique identifiers, or web links for publicly available datasets
- A list of figures that have associated raw data
- A description of any restrictions on data availability

The PDB accession codes for the coordinates of SARS-CoV-2 main protease are 7BAJ (apo), 7BAK (treated with ebselen) and 7BAL (treated with MR6-31-2).

Field-specific reporting

Life sciences study design

All studies must disclose on these points even when the disclosure is negative.

Sample size	Sample size estimation was not relevant for this study, as it does not report on a statistical evaluation of effects between two or more groups.
Data exclusions	Samples deemed to be technical failures were excluded. Two data points were verified to be extreme outliers and were therefore removed when calculating the IC50 values:<br>Fig. 1a (0.0625 µM ebselen with 0.01% Triton X-100, one of the three biological replicates)<br>Fig. 1f (5 µM MR6-31-2 with 0.01% Triton X-100, one of the three biological replicates).<br>Removal of these data points do not alter any conclusions made in this study.
Replication	To ensure reproducibility of experimental findings, each assay was performed at least three times to confirm the results. IC50 measurements (Fig. 1) were carried out with three biological replicates for each data point and these data were used to calculate mean values.
Randomization	Animals or human research participants were not involved in this study and, as such, samples were not randomized for the experiments.
Blinding	Animals or human research participants were not involved in this study and, as such, samples were not blinded for the experiments.

Reporting for specific materials, systems and methods

We require information from authors about some types of materials, experimental systems and methods used in many studies. Here, indicate whether each material, system or method listed is relevant to your study. If you are not sure if a list item applies to your research, read the appropriate section before selecting a response.

Materials & experimental systems

n/a	Involved in the study	
☒	☐	Antibodies
☐	☒	Eukaryotic cell lines
☒	☐	Palaeontology and archaeology
☒	☐	Animals and other organisms
☒	☐	Human research participants
☒	☐	Clinical data
☒	☐	Dual use research of concern

Methods

n/a	Involved in the study	
☒	☐	ChIP-seq
☒	☐	Flow cytometry
☒	☐	MRI-based neuroimaging

Eukaryotic cell lines

Policy information about cell lines

Cell line source(s)	African green monkey origin, Vero E6 from ATCC-1586
Authentication	All monkey cells were from ATCC with authentication. The authentication was performed by morphology check under microscopes and growth curve analysis
Mycoplasma contamination	We confirm that cell line has been tested negative for Mycoplasma contamination.
Commonly misidentified lines (See ICLAC register)	No commonly misidentified cell lines were used.
